# Attitudes towards Intimate Partner Violence against Women among Women and Men in 39 Low- and Middle-Income Countries

**DOI:** 10.1371/journal.pone.0167438

**Published:** 2016-11-28

**Authors:** Thach Duc Tran, Hau Nguyen, Jane Fisher

**Affiliations:** 1 Research and Training Centre for Community Development, Hanoi, Vietnam; 2 Jean Hailes Research Unit, School of Public Health and Preventive Medicine, Monash University, Melbourne, Victoria, Australia; Liverpool School of Tropical Medicine, UNITED KINGDOM

## Abstract

**Background:**

Violence against women perpetrated by an intimate partner (IPV) is prevalent in low- and middle-income countries (LAMIC). The aim was to describe the attitudes of women and men towards perpetration of physical violence to women by an intimate partner, in a large group of low- and middle-income countries.

**Methods and Findings:**

We used data from Round Four of the UNICEF Multiple Indicator Cluster Surveys. Attitudes towards IPV against women were assessed by a study-specific scale asking if ‘wife beating’ is justified in any of five circumstances.

Overall, data from 39 countries (all had data from women and 13 countries also had data from men) were included in the analyses. The proportions of women who held attitudes that ‘wife-beating’ was justified in any of the five circumstances varied widely among countries from 2.0% (95% CI 1.7;2.3) in Argentina to 90.2% (95% CI 88.9;91.5) in Afghanistan. Similarly, among men it varied from 5.0% (95% CI 4.0;6.0) in Belarus to 74.5% (95% CI 72.5;76.4) in the Central African Republic. The belief that ‘wife-beating’ is acceptable was most common in Africa and South Asia, and least common in Central and Eastern Europe and Latin America and the Caribbean. In general this belief was more common among people in disadvantaged circumstances, including being a member of a family in the lowest household wealth quintile, living in a rural area and having limited formal education. Young adults were more likely to accept physical abuse by a man of his intimate partner than those who were older, but people who had never partnered were less likely to have these attitudes.

**Conclusions:**

Violence against women is an international priority and requires a multicomponent response. These data provide evidence that strategies should include major public education programs to change attitudes about the acceptability of IPV against women, and that these should be addressed to women and girls as well as to boys and men.

## Introduction

Globally, the lifetime prevalence of experiencing violence perpetrated by an intimate partner (IPV) among women aged at least 15 years is estimated to be up to 30.0% (95% confidence interval (CI) 27.8;32.2) [1]. However, prevalence varies widely, including, among low- and middle-income countries (LMIC), from 13.7% in Cambodia to 70.9% in Ethiopia [2, 3]. IPV against men does occur but is less prevalent and less severe than that against women [4–7]. Almost all LMIC have patriarchal sociocultural and religious values and political systems which condone the violation of women’s rights [8]. In this study, we focus on attitudes toward physical violence perpetrated by an intimate partner against women.

Attitudes that IPV is acceptable and culturally normative are among the most significant factors associated with the likelihood of perpetration and social responses to perpetration [9–12]. Women who believe that IPV is acceptable and normative are more likely to blame themselves for the violence, and to experience long-term mental health problems, and less likely to report the problem to civil authorities or other family members [13]. The attitudes toward IPV of people other than the perpetrator or victim shape responses to the violence. People who regard IPV as a cultural norm tend to respond with less empathy and support to victims [14, 15]. Attitudes to and beliefs about IPV are therefore related not only to its prevalence but also to community responses to the violence.

Attitudes about IPV perpetrated against women are multifactorially determined. Social norms and beliefs about traditional gender roles shape attitudes and can be intergenerationally transmitted [16]. The patriarchal hegemony that is widely established in many LAMIC generally supports attitudes that women’s behaviours are the triggers for their partners’ violent behaviours and that men are justified to ‘discipline’ their wives for transgressions [17]. Experiencing or witnessing violence increases tolerance of IPV, and children who witness violence perpetrated by their fathers towards their mothers are more likely than those without this experience to believe that violence is appropriate and justifiable [18].

In South-Central Asia, the proportions of people in the general community who accept wife-beating are up to 57% among women and 56% among men [19]. In sub-Saharan Africa, these proportions were up to 74% among women and 62% among men [20]. There is in general however, a lack of studies examining these attitudes among both men and women across countries and regions.

The Multiple Indicator Cluster Surveys (MICS) are international household surveys initiated by the United Nations Children’s Fund (UNICEF) and have been implemented in up to five rounds in 108 LAMIC. The MICS’ primary goal is to monitor indicators of progress towards the Millennium Development Goals related to women’s and children’s health in low—and middle—income countries from the mid-1990s to 2015. Since Round Four, data about attitudes towards IPV against women have been collected from both women and men of reproductive age. These data can inform strategies to achieve the Global Sustainable Development Goals from 2016–2030, which have identified that gender equality and good health and wellbeing are priorities for every nation. The aims of this study were to estimate from data collected in MICS Round Four (http://mics.unicef.org/surveys) the proportions of women and men in a large number of LAMIC holding attitudes that IPV against women is acceptable; and to examine the associations among socio-demographic characteristics and these attitudes.

## Methods

### Participants

In the MICS Round Four (2010–2012), a large nationally representative sample of between 5,000 and 40 000 households was selected in each country using a multistage, cluster sampling technique [21]. All women and men aged 15 to 49 in these households were eligible to provide data in individual structured interviews.

### Variables

Attitudes about IPV against women were assessed by a single set of fixed response yes/no questions: if a husband is justified in hitting or beating his wife in any of the following circumstances: (1) she goes out without telling him, (2) she neglects the children, (3) she argues with him, (4) she refuses to have sex with him, (5) she burns the food. These circumstances reflect widespread stereotypes about women’s roles and responsibilities. The same questions were administered to women and men.

Household economic status was assessed using questions about household characteristics including the main materials of the dwelling’s floor, roof, and exterior walls; main type(s) of fuel used for cooking; source of drinking water; type of sanitation facility; and how many of 12 durable household assets (e.g. a refrigerator or a bicycle) were owned. An index of household wealth was constructed on the basis of these items using confirmation factor analysis [22, 23]. Demographic characteristics of women and men included age, education level, and marital status were collected using structured study-specific questions.

A nation’s Human Development Index (HDI), calculated by the United Nations Development Program (UNDP), is a proxy indicator which comprises the average achievement of a country in three basic dimensions of human development: a long and healthy life (average life expectancy), knowledge (average years of completed education) and a decent standard of living (proportion living above the international poverty line) [24]. Each country’s HDI was obtained from the UNDP’s Human Development Reports 2011 [25]. The HDI ranges from 0 (the lowest) to 1 (the highest) and is classified into very high (>0.790), high (>0.698 to 0.790), medium (>0.510 to 0.698), and low categories (≤ 0.510). The Gender Inequality Index (GII) is a composite measure reflecting inequality in achievement between women and men in three dimensions: reproductive health, empowerment and the labour market [25]. The GII ranges from 0 (the lowest inequality) to 1 (the highest inequality) and can be classified into high (0.60 or higher), medium (0.4 to <0.6), and low (< 0.4) inequality. National Expected Years of Schooling is the number of years of schooling that a child of school entrance age can expect to receive if prevailing patterns of age-specific enrolment rates persist throughout the child’s life.

All MICS data are collected in face-to-face individual structured interviews during home visits by national data collection teams. All questionnaires used in MICS can be found at the MICS website (http://mics.unicef.org/tools?round=mics4).

### Statistical analysis

Attitudes accepting of IPV were categorised as 1 if at least one of the five situations had been endorsed with a yes answer and as 0 if none had been endorsed. Prevalence and 95% confidence intervals (CI) of women and men having attitudes accepting IPV against women were calculated by country and are shown in bar charts for ease of visual comparisons. This prevalence was compared among countries on the basis of several country-level characteristics including the HDI, GII, and region. Because the number of countries in this study is relatively small, median values were used for the syntheses.

The associations between attitudes accepting of IPV against women and socio-demographic characteristics including living in an urban or rural area, age group (<25 years old versus ≥ 25 years), education level (up to 5 years of primary schooling versus 6 or more years), marital status (never partnered versus ever partnered), and household wealth index (poorest quintile versus all other quintiles) were examined using multiple logistic regressions for each country in separate analyses for women and men.

The sex disparity in the attitudes accepting of IPV against women in each country (for which data from both women and men are available) was determined by a multiple logistic regression controlling for socio-demographic characteristics.

All analyses were conducted by survey commands (SVY) in STATA Version 12 (StataCorp., Texas, United States of America) taking into account the MICS prescribed sampling weights and cluster and household effects.

### Ethical considerations

This study was approved by The Monash University Human Research Ethics Committee (CF15/4319–2015001861). All data used in this study are available for public use (download at http://mics.unicef.org/surveys) and completely de-identified.

## Results

### Sample

Overall, 39 countries had data from women and, among these, 13 countries also had data from men (S1 Table). In total, data from 439 614 women and 53 538 men were available for analyses. The numbers of female participants ranged from 1,253 in St Lucia to 55 194 in Iraq. The numbers of male participants ranged from 1,545 in Moldova to 9,951 in Laos. Among the 39 countries, there were 13 countries with a low HDI, 10 countries with an HDI in the medium range, and 13 countries with a high HDI.

### Prevalence

**Fig 1:**
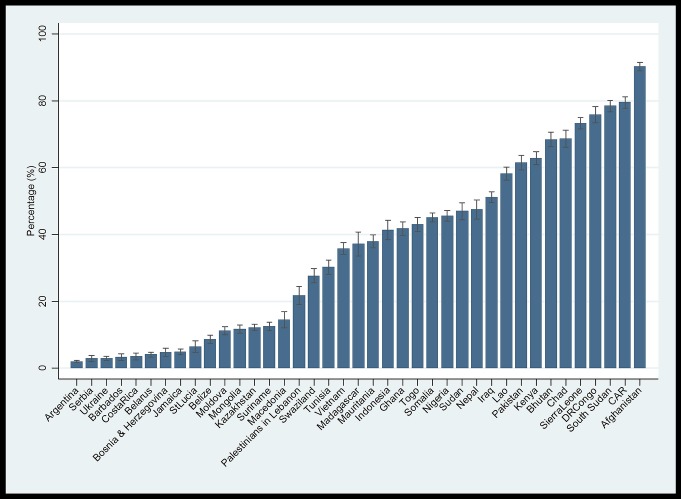
Percentages of women having attitudes accepting of a ‘husband beating his wife’ in any of five circumstances by country.

The prevalence of women having attitudes accepting of a husband beating his wife in at least one of the five situations varied widely among the 39 countries from 2.0% (95% CI 1.7;2.3) in Argentina to 90.2% (95% CI 88.9;91.5) in Afghanistan (Fig 1). The prevalence was higher in countries with lower HDI, higher GII, and lower national average years of schooling (Table 1). The prevalence varied among geographic regions. It was lowest in Central and Eastern Europe, the Commonwealth of Independent States and Latin America and the Caribbean, and highest in South Asia and West and Central Africa.

**Table 1:** Medians and ranges of the proportions of women having attitudes accepting of a husband ‘beating his wife’ among 39 countries by HDI, GII and region.

	Number of countries	Percetage of women having attitudes accepting of a ‘husband beating his wife’ (%)		
		Median^(a)^	Min	Max
**Human Development Index (HDI)** ^(b)^				
High (>0.698)	13	4.8	2.0	30.3
Medium (>0.510 to 0.698)	10	38.6	11.2	68.4
Low (0.510 or lower)	13	61.5	37.2	90.3
**Gender Inequality Index (GII)** ^(c)^				
Low (<0.4)	9	11.2	2.0	35.8
Medium (0.4 to <0.6)	11	41.8	4.9	68.4
High (0.6 or higher)	9	68.7	37.9	90.3
**National expected years of schooling**^(b)^				
High (> 12 years)	18	7.6	2.0	68.4
Medium (>9 to 12 years)	11	41.8	11.2	90.3
Low (9 years or lower)	7	61.5	37.9	79.6
**Region**				
Central and Eastern Europe	7	4.8	2.9	14.5
Latin America and Caribbean	7	4.9	2.0	12.5
Middle East and North Africa	4	38.7	21.8	51.2
Eastern and Southern Africa	5	45.1	27.6	78.5
West and Central Africa	8	57.2	37.9	79.6
East Asia and the Pacific	4	38.6	11.7	58.2
South Asia	4	64.9	47.5	90.3

^(a)^Median of the percetages among countries;

^(b)^ HDI rankings are not available for Palestinians in Lebanon, Somalia, and South Sudan;

^(c)^ GII rankings are not available for Belarus, Bosnia & Herzegovina, Madagascar, Nigeria, Palestinians in Lebanon, Serbia, Somalia, South Sudan, StLucia, Suriname.

**Fig 2:**
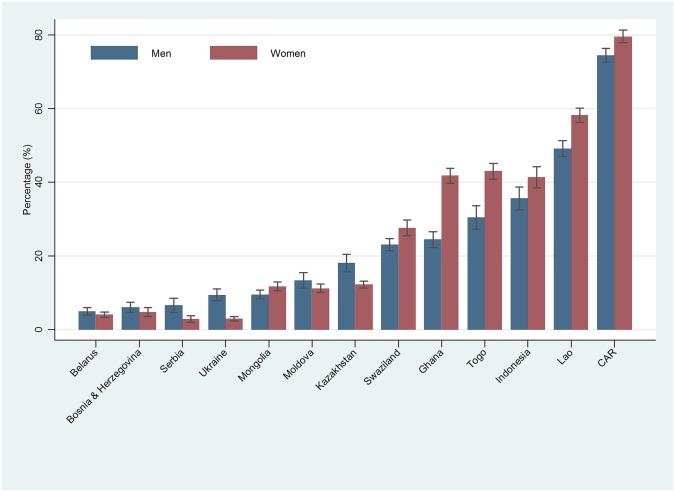
Percentages of women and men having attitudes accepting of a husband ‘beating his wife’ in any of five circumstances by country.

The range of the prevalence of men having attitudes accepting of a husband beating his wife among 13 countries ranged from 5.0% (95% CI 4.0;6.0) in Belarus to 74.5% (95% CI 72.5;76.4) in the Central African Republic (CAR) (Fig 2). Similar to the trend among women, the prevalence of these attitudes was higher in lower HDI and higher GII groups, including CAR and Laos and higher in Asian and African countries in comparison with countries in Central and Eastern Europe.

### Associations

The associations between socio-demographic characteristics and attitudes among women accepting of IPV against women are shown in Table 2. Overall, the associations are in the same direction in every country and reveal that living in a rural area, a household in the poorest quintile, being aged under 25 years, having limited education, or ever having been partnered were all associated with higher likelihood of attitudes accepting of IPV against women. However, none of these associations were significant in Madagascar and only one was significant in Costa Rica, Somalia, Chad, and Laos.

**Table 2:** Associations between socio-demographic characteristics and attitudes accepting of a ‘husband beating his wife’ among women in 39 countries.

Region/Country	Adjusted Odds Ratios (95% CI) ^(a)^				
	Living in a rural area	Living in the poorest quintile	Aged under 25 years	Having limited education^(c)^	Never partnered
**Central and Eastern Europe**					
Belarus	1.94	2.1	-^(b)^	1.56	-
Bosnia and Herzegovina	-	2.75	-	2.54	0.35
Kazakhstan	-	1.32	1.21	1.28	0.51
Macedonia	1.88	1.98	1.54	4.63	0.69
Moldova	-	1.93	-	1.77	-
Serbia	-	3.24	1.83	3.54	0.24
Ukraine	1.98	1.61	-	2.21	0.37
**Latin America and Caribbean**					
Argentina	-	1.83	1.6	3.42	-
Barbados	-	3.48	-	4.69	-
Belize	1.7	1.98	-	1.5	-
Costa Rica	-	-	-	3.1	-
Jamaica	1.8	-	2.14	2.92	-
St Lucia	0.56	1.86	2.61	3.34	-
Suriname	1.49	1.72	2.13	1.77	-
**Middle East & North Africa**					
Iraq	1.77	1.43	1.16	1.39	0.63
Palestinians in Lebanon	-	1.49	-	1.57	-
Sudan	-	2.32	1.45	1.81	0.88
Tunisia	1.82	1.63	0.86	1.97	0.85
**Eastern and Southern Africa**					
Kenya	1.83	-	1.44	1.84	0.56
Madagascar	-	-	-	-	-
Somalia	-	-	-	-	0.86
South Sudan	-	1.36	-	-	0.52
Swaziland	2.51	1.34	2.12	2.07	-
**West and Central Africa**					
Central African Republic	-	-	1.23	1.2	0.59
Chad	-	-	-	-	0.82
DR Congo	1.79	-	-	-	0.6
Ghana	1.76	1.58	1.93	1.75	0.66
Mauritania	1.41	1.21	-	1.46	0.83
Nigeria	1.32	1.15	1.12	-	0.63
Sierra Leone	1.83	-	-	1.88	0.51
Togo	1.25	-	1.32	1.33	0.73
**East Asia and the Pacific**					
Indonesia	-	1.4	1.51	1.3	-
Laos	-	-	-	-	0.82
Mongolia	2.19	-	-	1.58	-
Vietnam	1.49	1.48	-	1.57	0.75
**South Asia**					
Afghanistan	2.01	-	-	1.58	0.36
Bhutan	1.6	-	1.33	1.21	-
Nepal	-	1.35	-	1.5	0.62
Pakistan	1.54	0.72	-	-	0.6

Note: only significant odd ratios are presented in this table. For full information including 95% CIs please see S3 Table.

^(a)^Adjusted for each of the other sociodemographic characteristics and cluster effects;

^(b)^Omitted from the table because not significant;

^(c)^up to year 5 of primary schooling.

The associations were also tested among men by country (Table 3). Living in rural areas was associated with attitudes accepting of IPV against women among men in Asian and African countries except Laos and Togo, but not in Central and Eastern Europe except in Kazakhstan. In every country in Central and Eastern Europe and in 2 of 7 countries in Asia and Africa men living in the poorest households were more likely to justify IPV against women. There was a contrast in the association between age and attitudes accepting of IPV against women between countries in Central and Eastern Europe (men aged under 25 was less likely to have these attitudes than older men) and countries in Asia and Africa (men aged under 25 were more likely to have these attitudes). The association between having limited education and holding these attitudes was significant in countries in every area. Marital status was not commonly associated with these attitudes among men, a pattern that is opposite to the trend apparent among women. In Mongolia and the CAR men who had ever been partnered were significantly less likely to hold attitudes accepting of IPV against women.

**Table 3:** Associations between socio-demographic characteristics and attitudes accepting of a ‘husband beating his wife’ among men in 13 countries.

Region/Country	Adjusted odds ratio^(a)^ (95% CI)				
	Living in rural areas	Living in the poorest quintile	Under 25	Having limited education^(c)^	Never partnered
**Central and Eastern Europe**					
Belarus	-^(b)^	2.39	0.42	1.63	-
Bosnia & Herzegovina	-	2.25	-	2.89	-
Kazakhstan	1.52	1.68	-	-	-
Moldova	-	2.11	-	1.90	-
Serbia	-	2.05	-	2.75	-
Ukraine	-	1.92	0.40	-	-
**East Asia and the Pacific**					
Indonesia	1.35	2.91	1.34	-	-
Laos	-	-	-	1.45	-
Mongolia	1.47	-	-	1.45	1.57
**Africa**					
Central African Republic	1.37	-	1.42	-	1.64
Ghana	1.61	1.48	2.13	2.16	-
Swaziland	1.45	-	1.59	1.71	-
Togo	-	-	-	1.62	-

Note: only significant odd ratios are presented in this table. For full information including 95% CIs please see S2 Table.

^(a)^Adjusted for other characteristics in this table and cluster effects;

^(b)^Omitted from the table because not significant;

^(c)^up to year 5 of primary schooling.

The disparities between women and men in holding attitudes accepting of IPV against women in the 13 countries from which data were available were examined by logistic regression models taking into account the main socio-demographic characteristics (Table 4). In every country in Central and Eastern Europe, men were more likely to have these attitudes, but it was the opposite in every country in Asia and Africa in that women were more likely to have these attitudes.

**Table 4:** Adjusted odds ratios of having positive attitudes toward intimate partner violence against women among men (reference group: women) by country.

Region/Country	Adjusted odds ratio^(a)^ (95% CI)
**Central and Eastern Europe**	
Belarus	1.08 (0.33;1.43)
Bosnia & Herzegovina	1.61 (1.25;2.08)
Kazakhstan	1.57 (1.32;1.87)
Moldova	1.18 (0.96;1.46)
Serbia	3.63 (2.46;5.37)
Ukraine	3.50 (2.67;4.58)
**East Asia and the Pacific**	
Indonesia	0.79 (0.67;.093)
Laos	0.70 (0.64;0.77)
Mongolia	0.71 (0.62;0.82)
**Africa**	
Central African Republic	0.79 (0.70;0.90)
Ghana	0.47 (0.42;0.54)
Swaziland	0.74 (0.66;0.84)
Togo	0.63 (0.54;0.74)

^(a)^Adjusted for other characteristics including age, urban/rural, education level, household wealth index, marital status and cluster and household effects

## Discussion

This study analysed data collected from a very large sample of women and men in diverse low and middle income countries across the world in Round Four of the MICS (2010–2012). Responses to a defined set of unambiguous questions about attitudes toward IPV against women under a number of circumstances were used. These are well recognised as being the most appropriate data to make comparisons between sexes, countries and cultures [26]. We acknowledge that the questions only assessed attitudes towards physical abuse ‘wife beating’ and did not enquire into attitudes about sexual or emotional violence towards women and are therefore probably an underestimate of attitudes accepting of IPV against women.

There is considerable variation of the attitudes toward IPV against women at country and regional levels. The findings of this study are consistent with those found by Garcia-Moreno and her colleagues in the World Health Organization (WHO) Multi-Country Study of Violence Against Women that the prevalence of attitudes accepting of IPV against women is lower in Latin America and the Caribbean and Central and Eastern Europe than in South Asia and West and Central Africa [27]. The substantial heterogeneity of the attitudes among countries and regions appears to be linked to gender inequality. The GII of South Asian countries (median 0.57) and West and Central African countries (median 0.66) were considerably higher, indicating greater inequality, than these of Central and Eastern European countries (median GII 0.45) and Latin American and Caribbean countries (median 0.37). The data reveal the significant disparity of the proportion of people having attitudes accepting of IPV against women in high-GII countries (68.7% women) and low-GII countries (11.2% women). An analysis of data from 66 population-based surveys from 44 countries [28] found that the gender-related factors at the national level including norms related to male authority over female behaviour, the extent to which law and practice disadvantage women in access to land, property and other resources predict the population prevalence of IPV within the past 12 months. Gender inequality is rooted in the patriarchal system that is created and maintained by men. Patriarchy and the ideologies of male dominance have effects on laws, policy, criminal justice systems, and education that provide supportive conditions for the development and maintenance of attitudes justifying violence against women and girls [8, 29].

The substantial heterogeneity of the attitudes toward IPV against women among countries and regions appears also to be associated with country’s educational achievements. Heise et al. [28] found that country level education achievements have bivariate associations with the level of IPV but no association when analyses control for norms justifying wife beating that suggests the association between education achievements and the norms. Expected years of schooling of South Asian countries (median 10.8 years) and West and Central African countries (median 8.6 years) were also much lower than these of Central and Eastern European countries (median 13.6 years) and Latin America and Caribbean countries (median 13.5 years). These indicate that the more highly educated the population is, the less prevalent attitudes among women and men supportive of wife beating are. Our analysis show that 7.6% women in countries having on average at least 12 years of schooling agreed with IPV against women. However, in countries with only up to 9 years of schooling, on average 65.1% held this view. Lower average levels of education associated with higher gender-inequality and with norms about the acceptability of using violence as a tactic in relationship conflict and the expression of frustration or anger [29].

At the individual level, consistent with previous studies, it was found that living in rural areas, the poorest quintile of households and having a low educational level were associated with greater likelihood of justifying IPV against women among both women and men [9, 19, 30–33]. The acceptance rates of wife-beating among people living in rural areas was higher than among people living in urban areas even when household wealth and education level were taken into account indicating that social justification of IPV against women is in general higher in rural than urban areas. People in the lowest socioeconomic position (living in poverty and having low education) might be exposed in childhood to maltreatment, including witnessing violence perpetrated by their fathers against their mothers, and have fewer opportunities to know about rights to safety and global norms about gender equity and thereby be more likely to accept IPV against women.

Consistent with prior evidence, it was found that younger women in many countries were more likely to justify IPV against women than older women [9, 31, 32]. The association between age and acceptance of IPV was different in men. The direction of this association in men was similar to that among women in Asia and Africa, but the acceptance rate among young men in Latin American and Caribbean countries was lower than or similar to that among older men. This suggests that young men in Latin America and Caribbean are more open to gender equity. When controlling for age and other demographic characteristics, women who had never partnered were less likely to accept IPV against women than women who had ever been partnered. This association was found among women in most of the countries studied, apart from those in Latin America and the Caribbean. In men, this association was significant in only two countries: Mongolia and the Central African Republic, but in the opposite direction. These differences between sexes indicate that attitudes toward gender equity differ between men and women, and vary further with whether they are partnered or not. Women who have never partnered appear to have attitudes that are more accepting of gender equity than those who have ever partnered in the same age group and socioeconomic position, but the relationship is in the opposite direction or does not differ among men. The wide variation suggests that these attitudes are modifiable and that community norms and public discourse are powerful determinants of individual attitudes and values including about gender-based norms.

The data also confirm sex disparities in the acceptance of ‘wife beating’ found by others. Women in the Asian and African countries were more likely to justify IPV than men when controlling for other demographic characteristics. Previous studies in Vietnam [34], an Asian country, and sub-Saharan Africa [20] also found that attitudes accepting of IPV against women were more prevalent among women than men. Those countries have the highest GII. In these settings, men are better educated, more likely to be employed in income-generating work than women and, as a result, have more access to information. The findings can suggest the crucial role of gender equity and women empowerment in efforts to reduce violence against women and girls.

Overall, several country level indices including GII, GDP, and education achievements appear to be indicators of the prevalence of attitudes supportive of IPV against women. The data indicate that increased attention and greater efforts from government and non-government organisations in the countries where acceptance is most widespread is an urgently-needed first step to reduce these attitudes. Policies aiming to improve gender equity, economic status, and education level at the country level can reduce the prevalence of the attitudes supportive of violence against women and girls in general, and IPV against women in particular. At the individual level, several sub-groups warrant a targeted focus, including women, young people, people living in rural areas, living in the poorest families, having low education levels, and ever having been partnered. Future studies can test if providing information and education on IPV for recently-married couples who are living in poor areas is effective for preventing IPV [35]. However, the magnitudes of the differences of the attitudes toward IPV against women among groups by socio-demographic characteristics are not huge in most countries and indicate that universal interventions are warranted. Future studies should examine if this kind of intervention can help to change social norms about gender equality and have wider effects. The formal education sector can be a channel to educate young people about respectful relationships. There is the need for intervention programs in schools for adolescents and young adults. The informal sector also needs to play an important role in many countries to reach young people not in schools and improve participation in formal education. It is warranted to investigate if programs aiming at improving the schooling rate and household economic status can reduce domestic violence against women and girls in the future studies.

In conclusion, the acceptance rates of IPV vary widely across countries, but are most common in South Asia and Africa. HDI, GII, and country education level are significantly associated with the rate of attitudes accepting of IPV against women across countries. In the countries where the acceptance of IPV against women is widespread, women are more likely to justify IPV than men, but the opposite association is found in the countries with generally low acceptance rates (Central and Eastern Europe). Rural areas, the poorest families, young and poorly educated people are most likely to justify IPV against women in most LAMIC countries and are clear targets for ameliorative efforts. Given the consequences of these negative attitudes, these data indicate that countering attitudes accepting of violence against women should be national priorities towards the achievement of gender equality and good health and wellbeing, the key Global Sustainable Development Goals.

## Supporting Information

S1 Table Number of participants by countries. (DOCX)

S2 Table Associations between socio-demographic characteristics and attitudes accepting of a ‘husband beating his wife’ among women in 39 countries. (DOCX)

S3 Table Associations between socio-demographic characteristics and attitudes accepting of a ‘husband beating his wife’ among men in 13 countries. (DOCX)
