# Supplementary material for: Attitudes towards Intimate Partner Violence against Women among Women and Men in 39 Low- and Middle-Income Countries
Source: PLoS One. 2016 Nov 28;11(11):e0167438. doi: 10.1371/journal.pone.0167438 (PMC5125706; doi:10.1371/journal.pone.0167438)
Supplement: S1 Table — (DOCX) [file pone.0167438.s001.docx]

**S1 Table. Number of participants by countries**

| Country | Region | Women | | Men | |
| --- | --- | --- | --- | --- | --- |
|  |  | Number of participants | Age  Mean (SD) | Number of participants | Age  Mean (SD) |
| Afghanistan | South Asia | 21,290 | 27.1 (9.3) | - | - |
| Argentina | Latin America and Caribbean | 21,660 | 30.7 (9.9) | - | - |
| Barbados | Latin America and Caribbean | 1,543 | 32.6 (9.9) | - | - |
| Belarus | Central and Eastern Europe | 5,745 | 31.8 (8.7) | 2,769 | 36.8 (12) |
| Belize | Latin America and Caribbean | 4,096 | 29.3 (9.8) | - | - |
| Bhutan | South Asia | 14,018 | 30.2 (9.3) | - | - |
| Bosnia and Herzegovina | Central and Eastern Europe | 4,446 | 31.7 (9.1) | 4,353 | 32.6 (9.2) |
| Central African Republic | West and Central Africa | 11,510 | 28.1 (9.2) | 5,311 | 31.7 (11.5) |
| Chad | West and Central Africa | 14,369 | 27.7 (9) | - | - |
| Costa Rica | Latin America and Caribbean | 5,084 | 30.2 (9.8) | - | - |
| DR Congo | West and Central Africa | 12,853 | 28.2 (9.4) | - | - |
| Ghana | West and Central Africa | 11,921 | 30.2 (9.9) | 3,928 | 32.2 (12.7) |
| Indonesia | East Asia and the Pacific | 5,499 | 30.5 (9.5) | 5,304 | 31.2 (9.9) |
| Iraq | Middle East & North Africa | 55,194 | 28.6 (9.6) | - | - |
| Jamaica | Latin America and Caribbean | 5,032 | 30.8 (10.2) | - | - |
| Kazakhstan | Central and Eastern Europe | 14,014 | 31.7 (10.1) | 3,628 | 36.9 (11.7) |
| Kenya | Eastern and Southern Africa | 6,729 | 28.2 (9.2) | - | - |
| Laos | East Asia and the Pacific | 22,476 | 29.6 (9.9) | 9,951 | 29.6 (10) |
| Macedonia | Central and Eastern Europe | 3,831 | 31.4 (9.7) | - | - |
| Madagascar | Eastern and Southern Africa | 2,897 | 28 (9.8) | - | - |
| Mauritania | West and Central Africa | 12,755 | 28.6 (9.5) | - | - |
| Moldova | Central and Eastern Europe | 6,000 | 31.5 (10) | 1,545 | 31.2 (10.3) |
| Mongolia | East Asia and the Pacific | 10,489 | 31.7 (9.8) | 5,442 | 32.4 (10.9) |
| Nepal | South Asia | 7,372 | 29 (9.5) | - | - |
| Nigeria | West and Central Africa | 30,772 | 29 (9.3) | - | - |
| Pakistan | South Asia | 17,732 | 28.1 (9.5) | - | - |
| Palestinians in Lebanon | Middle East & North Africa | 5,453 | 30.1 (10.2) | - | - |
| Serbia | Central and Eastern Europe | 5,385 | 31.8 (8.7) | 1,583 | 23.6 (4.3) |
| Sierra Leone | West and Central Africa | 13,359 | 28.3 (8.8) | - | - |
| Somalia | Eastern and Southern Africa | 11,357 | 27.3 (8.8) | - | - |
| South Sudan | Eastern and Southern Africa | 9,069 | 28.8 (8.5) | - | - |
| St Lucia | Latin America and Caribbean | 1,253 | 31.4 (10.4) | - | - |
| Sudan | Middle East & North Africa | 17,173 | 28.2 (9) | - | - |
| Suriname | Latin America and Caribbean | 6,290 | 30.8 (10) | - | - |
| Swaziland | Eastern and Southern Africa | 4,688 | 28.1 (9.6) | 4,179 | 29.2 (11.6) |
| Togo | West and Central Africa | 6,376 | 29.4 (9.5) | 1,925 | 31.3 (12.4) |
| Tunisia | Middle East & North Africa | 10,215 | 30.6 (9.9) | - | - |
| Ukraine | Central and Eastern Europe | 8,006 | 31.5 (8.8) | 3,620 | 32.2 (8.9) |
| Vietnam | East Asia and the Pacific | 11,663 | 31.5 (9.9) | - | - |
