# Supplementary material for: Attitudes towards Intimate Partner Violence against Women among Women and Men in 39 Low- and Middle-Income Countries
Source: PLoS One. 2016 Nov 28;11(11):e0167438. doi: 10.1371/journal.pone.0167438 (PMC5125706; doi:10.1371/journal.pone.0167438)
Supplement: S2 Table — (DOCX) [file pone.0167438.s002.docx]

**S2 Table. Associations between socio-demographic characteristics and attitudes accepting of a ‘husband beating his wife’ among women in 39 countries**

| Country | Adjusted odds ratio^(a)^ (95% CI) | | | | |
| --- | --- | --- | --- | --- | --- |
|  | Living in rural areas | Living in the poorest  quintile | Under 25 | Having a low education level | Never partnered |
| Belarus | 1.94 (1.29;2.91) | 2.10 (1.32;3.34) | 0.57 (0.31;1.05) | 1.56 (1.04;2.34) | 0.91 (0.47;1.77) |
| Bosnia & Herzegovina | 0.72 (0.37;1.40) | 2.75 (1.80;4.20) | 0.99 (0.52;1.89) | 2.54 (1.63;3.95) | 0.35 (0.16;0.73) |
| Kazakhstan | 1.16 (0.97;1.39) | 1.32 (1.07;1.62) | 1.21 (1.01;1.46) | 1.28 (1.12;1.47) | 0.51 (0.42;0.62) |
| Macedonia | 1.88 (1.31;2.70) | 1.98 (1.45;2.71) | 1.54 (1.08;2.2) | 4.63 (3.37;6.35) | 0.69 (0.48;0.99) |
| Moldova | 1.20 (0.94;1.53) | 1.93 (1.46;2.53) | 0.98 (0.73;1.32) | 1.77 (1.43;2.2) | 1.00 (0.73;1.35) |
| Serbia | 1.89 (0.96;3.74) | 3.24 (1.59;6.60) | 1.83 (1.01;3.35) | 3.54 (1.92;6.53) | 0.24 (0.11;0.53) |
| Ukraine | 1.98 (1.17;3.33) | 1.61 (1.01;2.56) | 0.97 (0.59;1.60) | 2.21 (1.46;3.33) | 0.37 (0.20;0.68) |
| Indonesia | 0.95 (0.74;1.21) | 1.4 (1.09;1.79) | 1.51 (1.28;1.79) | 1.30 (1.12;1.51) | 0.97 (0.79;1.21) |
| Laos | 1.09 (0.91;1.30) | 0.9 (0.79;1.02) | 1.00 (0.92;1.08) | 0.92 (0.81;1.05) | 0.82 (0.74;0.90) |
| Mongolia | 2.19 (1.70;2.83) | 1.07 (0.83;1.39) | 1.04 (0.88;1.23) | 1.58 (1.29;1.93) | 1.12 (0.92;1.36) |
| Vietnam | 1.49 (1.29;1.73) | 1.48 (1.29;1.71) | 1.11 (0.98;1.26) | 1.57 (1.37;1.79) | 0.75 (0.65;0.88) |
| Kenya | 1.83 (1.43;2.34) | 1.04 (0.89;1.22) | 1.44 (1.24;1.66) | 1.84 (1.59;2.12) | 0.56 (0.48;0.66) |
| Somalia | 1.05 (0.92;1.21) | 0.99 (0.85;1.16) | 0.99 (0.89;1.09) | 0.99 (0.90;1.09) | 0.86 (0.76;0.96) |
| South Sudan | 0.97 (0.75;1.24) | 1.36 (1.11;1.67) | 0.96 (0.83;1.12) | 1.00 (0.85;1.19) | 0.52 (0.43;0.63) |
| Swaziland | 2.51 (1.96;3.21) | 1.34 (1.05;1.71) | 2.12 (1.79;2.50) | 2.07 (1.77;2.41) | 1.17 (0.97;1.40) |
| Iraq | 1.77 (1.54;2.02) | 1.43 (1.28;1.61) | 1.16 (1.08;1.24) | 1.39 (1.27;1.52) | 0.63 (0.58;0.68) |
| Lebanon(Palestinians) | 1.18 (0.86;1.62) | 1.49 (1.16;1.91) | 1.07 (0.90;1.26) | 1.57 (1.33;1.86) | 0.93 (0.77;1.13) |
| Tunisia | 1.82 (1.47;2.25) | 1.63 (1.33;2.00) | 0.86 (0.75;0.98) | 1.97 (1.62;2.39) | 0.85 (0.75;0.97) |
| Afghanistan | 2.01 (1.53;2.65) | 0.76 (0.50;1.16) | 0.93 (0.82;1.05) | 1.58 (1.31;1.91) | 0.36 (0.29;0.43) |
| Bhutan | 1.60 (1.27;2.01) | 0.89 (0.73;1.08) | 1.33 (1.17;1.51) | 1.21 (1.06;1.38) | 0.88 (0.76;1.02) |
| Nepal | 0.82 (0.62;1.07) | 1.35 (1.10;1.67) | 0.96 (0.82;1.12) | 1.5 (1.27;1.76) | 0.62 (0.51;0.75) |
| Pakistan | 1.54 (1.23;1.92) | 0.72 (0.59;0.89) | 0.93 (0.81;1.05) | 0.98 (0.86;1.12) | 0.60 (0.52;0.69) |
| Argentina |  | 1.83 (1.25;2.67) | 1.60 (1.15;2.23) | 3.42 (2.42;4.83) | - |
| Barbados | 0.68 (0.35;1.29) | 3.48 (2.04;5.92) | 1.08 (0.57;2.05) | 4.69 (1.71;12.83) | 1.57 (0.70;3.51) |
| Belize | 1.70 (1.23;2.35) | 1.98 (1.44;2.71) | 1.23 (0.95;1.58) | 1.50 (1.15;1.96) | 1.23 (0.93;1.62) |
| Costa Rica | 0.79 (0.45;1.38) | 1.92 (0.90;4.09) | 0.80 (0.44;1.44) | 3.10 (1.47;6.52) | 1.40 (0.81;2.42) |
| Jamaica | 1.80 (1.13;2.87) | 1.32 (0.95;1.83) | 2.14 (1.54;2.98) | 2.92 (1.79;4.76) | 0.80 (0.55;1.15) |
| St Lucia | 0.56 (0.33;0.96) | 1.86 (1.11;3.12) | 2.61 (1.57;4.34) | 3.34 (1.42;7.82) | 1.1 (0.65;1.88) |
| Suriname | 1.49 (1.17;1.90) | 1.72 (1.34;2.21) | 2.13 (1.71;2.66) | 1.77 (1.35;2.32) | 0.82 (0.67;1.01) |
| Central African Republic | 1.05 (0.86;1.29) | 1 (0.83;1.2) | 1.23 (1.09;1.38) | 1.2 (0.91;1.58) | 0.61 (0.5;0.75) |
| Chad | 0.96 (0.76;1.21) | 1.06 (0.86;1.30) | 0.93 (0.83;1.03) | 0.84 (0.71;1.01) | 0.82 (0.68;0.98) |
| DR Congo | 1.79 (1.37;2.34) | 1.21 (0.95;1.55) | 1.11 (0.97;1.28) | 0.90 (0.75;1.09) | 0.6 (0.5;0.72) |
| Ghana | 1.76 (1.48;2.11) | 1.58 (1.35;1.84) | 1.93 (1.61;2.30) | 1.75 (1.5;2.040) | 0.66 (0.56;0.78) |
| Nigeria | 1.32 (1.15;1.53) | 1.15 (1.01;1.30) | 1.12 (1.02;1.24) | 1.05 (0.95;1.17) | 0.63 (0.56;0.71) |
| Sierra Leone | 1.83 (1.58;2.11) | 1.18 (0.98;1.43) | 1.11 (0.97;1.26) | 1.88 (1.66;2.14) | 0.51 (0.45;0.59) |
| Togo | 1.25 (1.03;1.51) | 1.01 (0.84;1.21) | 1.32 (1.12;1.56) | 1.33 (1.15;1.52) | 0.73 (0.6;0.89) |
| Madagascar | 0.85 (0.51;1.41) | 0.86 (0.66;1.11) | 0.98 (0.79;1.21) | 0.76 (0.58;0.99) | 1.01 (0.78;1.29) |
| Sudan | 1.21 (1.00;1.46) | 2.32 (1.90;2.84) | 1.45 (1.31;1.60) | 1.81 (1.60;2.05) | 0.88 (0.78;1) |
| Mauritania | 1.41 (1.19;1.67) | 1.21 (1.02;1.44) | 1.01 (0.92;1.10) | 1.46 (1.29;1.65) | 0.83 (0.74;0.94) |
