# Supplementary material for: Attitudes towards Intimate Partner Violence against Women among Women and Men in 39 Low- and Middle-Income Countries
Source: PLoS One. 2016 Nov 28;11(11):e0167438. doi: 10.1371/journal.pone.0167438 (PMC5125706; doi:10.1371/journal.pone.0167438)
Supplement: S3 Table — (DOCX) [file pone.0167438.s003.docx]

**S3 Table. Associations between socio-demographic characteristics and attitudes accepting of a ‘husband beating his wife’ among men in 13 countries**

| Country | Adjusted odds ratio^(a)^ (95% CI) | | | | |
| --- | --- | --- | --- | --- | --- |
|  | Living in rural areas | Living in the poorest  quintile | Under 25 | Having a low education level | Never partnered |
| Belarus | 1.14 (0.68;1.93) | 2.39 (1.38;4.13) | 0.42 (0.18;0.99) | 1.63 (1.02;2.59) | 0.94 (0.49;1.83) |
| Bosnia & Herzegovina | 0.69 (0.39;1.22) | 2.25 (1.46;3.48) | 1.03 (0.57;1.85) | 2.89 (1.83;4.57) | 0.78 (0.48;1.26) |
| Kazakhstan | 1.52 (1.07;2.16) | 1.68 (1.24;2.27) | 1.30 (0.88;1.94) | 1.24 (0.96;1.60) | 0.81 (0.57;1.15) |
| Moldova | 1.08 (0.74;1.58) | 2.11 (1.35;3.28) | 0.93 (0.56;1.56) | 1.90 (1.28;2.83) | 1.01 (0.62;1.63) |
| Serbia | 1.93 (0.98;3.80) | 2.05 (1.05;4.01) | 1.26 (0.60;2.61) | 2.75 (1.41;5.38) | 0.69 (0.30;1.60) |
| Ukraine | 1.34 (0.88;2.04) | 1.92 (1.32;2.80) | 0.40 (0.24;0.66) | 1.27 (0.85;1.92) | 0.72 (0.45;1.17) |
| Indonesia | 1.35 (1.04;1.75) | 2.91 (2.18;3.89) | 1.34 (1.09;1.65) | 1.19 (0.99;1.44) | 1.04 (0.83;1.31) |
| Laos | 0.94 (0.76;1.16) | 1.02 (0.87;1.19) | 1.06 (0.93;1.21) | 1.45 (1.17;1.81) | 0.96 (0.83;1.11) |
| Mongolia | 1.47 (1.07;2.02) | 1.02 (0.74;1.41) | 0.74 (0.55;1.01) | 1.45 (1.14;1.85) | 1.57 (1.16;2.11) |
| Swaziland | 1.45 (1.19;1.78) | 1.05 (0.84;1.33) | 1.59 (1.25;2.01) | 1.71 (1.46;2.02) | 1.21 (0.95;1.53) |
| Central African Republic | 1.37 (1.11;1.69) | 1.07 (0.85;1.34) | 1.42 (1.11;1.82) | 1.18 (0.98;1.43) | 1.64 (1.23;2.18) |
| Ghana | 1.61 (1.24;2.08) | 1.48 (1.17;1.88) | 2.13 (1.58;2.87) | 2.16 (1.63;2.86) | 1.00 (0.72;1.38) |
| Togo | 1.39 (0.98;1.97) | 1.21 (0.87;1.67) | 0.96 (0.67;1.39) | 1.62 (1.21;2.16) | 1.39 (0.97;1.99) |
